# A survey of the impact of owning a service dog on quality of life for individuals with physical and hearing disability: a pilot study

**DOI:** 10.1186/s12955-017-0640-x

**Published:** 2017-03-29

**Authors:** Sophie S. Hall, Jessica MacMichael, Amy Turner, Daniel S. Mills

**Affiliations:** University of Lincoln, School of Life Sciences, Lincoln, UK

**Keywords:** Service dog, Health, Hearing disability, Physical disability, Quality of life

## Abstract

**Background:**

Quality of life refers to a person’s experienced standard of health, comfort and happiness and is typically measured using subjective self-report scales. Despite increasing scientific interest in the value of dogs to human health and the growing demand for trained service dogs, to date no research has reported how service dogs may affect client perceptions of quality of life.

**Method:**

We compared quality of life scores on the 16 item Flanagan quality of life scale from individuals who owned a trained service dog with those who were eligible to receive a dog, but did not yet have one (waiting list control). Data were analysed separately from two groups; those with a service dog trained for individuals with physical disabilities (with physical service dog: *n* = 72; waiting for a service dog: *n* = 24; recruited from Dogs for Good database) and those with a hearing service dog (with hearing service dog = 111; waiting for a service dog = 30; recruited from Hearing Dogs for Deaf People database).

**Results:**

When controlling for age and gender individuals scored higher on total quality of life scores if they owned a service dog or a hearing service dog, but this was only statistically significant for those with a service dog. Both groups (physical service dog and hearing service dog) scored significantly higher on items relating to health, working, learning and independence if they owned a service dog, in comparison to those on the waiting list. Those with a physical service dog also scored significantly higher on items relating to recreational activities (including items relating to reading/listening to music, socialising, creative expression), and those involving social interactions (including items relating to participating in organisations, socialising, relationship with relatives). Additionally, those with a physical service dog scored higher on understanding yourself and material comforts than those on the waiting list control. In contrast, those with a hearing service dog appeared to receive fewer benefits on items relating to social activities.

**Conclusions:**

Owning a service dog can bring significant specific and potentially general benefits to the quality of life of individuals with physical disabilities and hearing impairments. These benefits may have considerable implications for individuals with disabilities, society and the economy by promoting independence, learning and working abilities.

## Background

Quality of life can be defined as an individual’s experienced standard of health, comfort, and happiness [1]. It is a broad concept that describes both negative and positive aspects of life. Quality of life is multidimensional; it includes mental health, physical health and social concepts. It is typically assessed through self-report scales [2] using condition specific (e.g. [3–5]) or general health related quality of life (e.g. [6]). Assessing quality of life can be beneficial in medical practice; allowing an individual to self-assess their health status has shown to be a strong indicator and predictor of mortality [7, 8]. Additionally, by assessing the subjective effect of living with a disease or disability it is possible to identify high risk groups at which to target health interventions, to improve quality of life and satisfaction of intervention protocols [9].

Disabilities can take many different forms, from physical and mobility impairments to sensory deficiencies and neurodevelopmental delays. Living with a disability itself may not decrease an individual’s quality of life as much as their perceptions of their ability to cope with their needs and situation [10]. Indeed, an individual’s perception of their disability affects their occupation, social life and their sense of independence [10, 11]. Therefore, whilst interventions that reduce the impact of the physical consequences of living with a disability may improve perceived quality of life, it is also important that strategies are considered which promote the ability of the individual to adjust psychologically to their situation [10].

There is growing recognition of the value of animal companionship to human health [12]. The numbers and roles of service dogs are expanding globally [13]. Indeed, there is increased demand for trained service dogs from organisations such as Dogs for Good and Hearing Dogs for Deaf People. Service dogs can be applied for when an individual is living with a disability. These dogs have been trained in remarkable ways to not only provide sensory or motor assistance, but also to look after an owner’s health and wellbeing [14]. Guide dogs, hearing dogs, and dogs for individuals with physical disabilities, enhance an owner’s mobility and independence [15]. Service dogs can be trained to retrieve items for owners, open doors, undress owners, operate light switches and pull wheelchairs, amongst other behaviours. Hearing service dogs are trained to alert individuals to important sounds, such as the telephone and doorbell, fire alarms and babies crying. The benefits of owning a service dog have been studied in terms of what services these dogs can provide for an owner - how they improve mobility and ease tasks [16]. However, dogs are also known to provide significant psychological benefits to an owner, such as increased social acknowledgement from the public [17]. Service dogs allow their owners to gain a larger degree of freedom and enhance their ability to partake in everyday outings or tasks that may otherwise have been a struggle, or impossible, alone. The independence a service dog can bring to an owner can mean better social integration, increased social acknowledgement, occupational changes, increased positive affect, and boosted confidence and self-esteem [18–21]. Owning a service dog has been shown to enhance psychological wellbeing and worth [16] and this is likely to positively impact an owner’s perception of their overall quality of life. Despite this, little research has considered the specific impact owning a service dog may have on perceived quality of life and its various facets, with only qualitative investigations in this area to date [22].

The aim of this project was to assess the impact that owning a service dog (for physical assistance or hearing assistance) may have on a client’s perceived quality of life in comparison to the quality of life scores obtained from individuals who need a service dog but do not yet own one. It was predicted that there would be a significant difference in quality of life scores between those ‘with service dogs’ in comparison to those ‘waiting for a service dog’. We recognised that individuals who were eligible to receive a service dog for physical needs and individuals who were eligible to receive a hearing dog would face very different demands and challenges to each other, therefore analyses were conducted separately for these two groups of participants.

## Methods

### Participants

All participants were self-recruited from the databases held by the United Kingdom (UK) charitable organisations Dogs for Good (for the physical service dog owners and controls) and Hearing Dogs for Deaf People (for the hearing service dog owners and controls). Given that service dogs work with a range of individuals, across disabilities, the only stipulation set on participating in the study was that the individual was over 18 years and met the criteria for receiving a service dog set by Dogs for Good and Hearing Dogs for Deaf People, as appropriate (see Table 1 for information on age and gender of the participants).

**Table 1:** The distribution of age category and gender across the groups

	Physical Service Dog		Hearing Service Dog	
	With Dog (*n* = 72)	Waiting List (*n* = 24)	With Dog (*n* = 111)	Waiting List (*n* = 30)
Age				
18–25 years	1 (1.4%)	4 (16.7%)	1 (0.9%)	1 (3.3%)
25–35 years	4 (5.6%)	2 (8.3%)	4 (3.6%)	2 (6.7%)
35–45 years	14 (19.4%)	1 (4.2%)	17 (15.3%)	3 (10%)
45–55 years	21 (29.2%)	9 (37.5%)	34 (30.6%)	7 (23.3%)
55+ years	32 (44.4%)	8 (33.3%)	55 (49.5%)	17 (56.7%)
Gender				
Men	14 (19.4%)	5 (20.8%)	19 (17.1%)	13 (43.3%)
Women	58 (80.6%)	19 (79.2%)	92 (82.9%)	17 (56.7%)

### Ethics, Consent and Permissions

All testing procedures complied with the British Psychological Society (BPS) Ethics Code of Conduct [23] and ethical approval was obtained from the designated authority of the College of Science Ethics Committee, University of Lincoln. Participants gave written, fully-informed consent to participate in the study and for their data to be reported anonymously.

### Design and Materials

We used a case control survey design to collect data from individuals who currently owned a service dog and those who were waiting to receiving a service dog (control). The first part of the questionnaire asked the client to state their age, gender, and service dog status along with an open question asking clients to give a brief description of their disability. The second part of the questionnaire formed the quality of life assessment. We used a 16-item adaptation of the original 15 item Flanagan Quality of Life Scale (QOLS) which is suitable for use with participants living with chronic conditions [24, 25]. The additional 16th item assesses independence. Independence was thought to be important in this study because service dogs are often applied for in the belief that they increase their owner’s independence and ability to do more without assistance from others. Construct validity of the 16 item Flanagan QOLS has been documented [26]. Respondents are asked to read each item and mark the number that best describes how satisfied they are at the present time. Example items include “Health - being physically fit and vigorous” and “Helping and encouraging others, volunteering, giving advice”. We used the seven-point scale for scoring each of the 16 items (1 = Terrible, 4 = Mixed, 7 = Delighted). The seven-point scale is thought to be more sensitive and less negatively biased than the five-point scale originally used [27]. Although it has been suggested that a three-factor structure exists for this scale the loading of each item on the factors differs depending on participant gender and well-being status [26]. Therefore, given the relatively small numbers tested in this study it was considered inappropriate to assess responses in relation to three factors. Instead the data analysis procedures followed that outlined in the original design of the scale, which includes consideration of individual items and a total quality of life score, which is obtained by summing the individual scores from each item [25].

### Procedure

Questionnaires were distributed to contacts through the postal system and via email. Pre-paid envelopes were provided to individuals who wished to use the postal option. Participants were also offered the opportunity to complete the questionnaire over the telephone if this was more convenient; one participant responded using this method (with a physical service dog). A reminder message was sent out after 2 weeks if no response had been returned. Participants were given 4 weeks to respond to the questionnaire before responses were collated.

#### Physical Service Dogs

In total 208 Dogs for Good clients were contacted to take part in the study. Participants were asked to return the forms if they wished to be involved in the study. A total of 96 participants responded to the questionnaire (46.2% response rate). From these 96 responses 72 participants had been trained to work with, and currently lived with a service dog (53% response rate); 24 participants had qualified for and were waiting for their service dog, or they had contacted the charity in order to be assessed to see if they qualified for a service dog (89% response rate). Participants were diagnosed with a range of impairments including: Arthritis (*n* = 7 with dog; *n* = 2 waiting for dog); wheelchair user due to spinal injury/virus/disease (*n* = 21 = with dog; *n* = 8 waiting for dog), Multiple Sclerosis (MS) (*n* = 18 with dog; *n* = 5 waiting for dog), impairment from disease/virus (e.g. polio) (*n* = 6 with dog; *n* = 1 waiting for dog), stroke (*n* = 3 with dog; *n* = 2 waiting for dog) and Ehlers danslos syndrome (*n* = 2 with dog; *n* = 2 waiting for dog). Other impairments mentioned by the ‘with dog’ group included muscular injury/wastage (*n* = 4), spondyloptosis (*n* = 1), neurological/brain disorders (*n* = 3), arthrogryposis (*n* = `), cerebral palsy (*n* = 1) and spina bifida (*n* = 1). Other impairments mentioned by those on the waiting list control included, seizures (*n* = 1), dystonia (*n* = 1), and scleroderma (*n* = 1). Some participants in both groups chose not to disclose their disability (*n* = 4 with dog; *n* = 1 waiting for dog).

#### Hearing Service Dog

In total 689 clients were emailed, 22 of these emails were returned undelivered leaving 667 contacts made; 565 of these owned a hearing dog and 102 were on the waiting list to receive one. A total of 260 individuals responded to the questionnaire (39% response rate). However, only 141 (21% response rate) had completed the questionnaire, of which 111 participants currently lived with a hearing service dog (20% response rate) and 30 were waiting to receive their dog (29% response rate).

### Data Analysis

Given the current lack of consensus on whether the Flanagan Quality of Life Scale is best measured as a three-factor scale, or total Quality of Life scores [26] we completed a staged analysis. In the first stage, we conducted a univariate Analysis of Co-Variance (ANCOA), with total Quality of Life Scores as the dependent variable, Service Dog Status as the fixed factor and Age and Gender as co-variates, to control for effects of these variables on quality of life scores. In the second stage, we conducted a multivariate ANCOVA, following the same approach as above, but with the 16 individual items included as dependent variables. All post-hoc analyses for significant (*p* < 0.05) effects were conducted using Bonferroni corrections and partial eta squared effect sizes (*η*_*p*_^*2*^), are reported as appropriate for ANCOVA. All values are reported in Table 2 (physical service dogs) and Table 3 (hearing service dogs) for total group (corrected after controlling for the effects of age and gender). Our sample was not large enough to complete reliable statistical analysis separately for men and women, however, we report descriptive data separately for these groups to power future studies.

**Table 2:** Descriptive data for quality of life scores across gender, and separately for men and women who own a physical service dog, and those on a waiting list: Data reported is means ± standard error means and partial eta squared (η_p_^2^) data

	Across Gender			Men			Women		
	With Dog (*n* = 72)	Waiting List (*n* = 24)	Power × (η_p_^2^)	With Dog (*n* = 14)	Waiting List (*n* = 5)	Power (η_p_^2^)	With Dog (*n* = 58)	Waiting List (*n* = 19)	Power (η_p_^2^)
Total Quality of Life	**80.72 ± 1.68^a^	**64.27 ± 2.94	.20	81.34 ± 4.61	57.23 ± 8.14	.28	80.87 ± 1.81	65.23 ± 3.22	.20
1. Material comforts home, food, conveniences, financial security	*5.54 ± 0.13^b^	*4.94 ± 0.23	.05	5.79 ± 0.3	2.65 ± 1.01	.02	5.51 ± 0.12	4.79 ± 0.28	.06
2. Health - being physically fit and vigorous	**3.57 ± 0.19	**2.40 ± 0.33	.09	3.98 ± 0.57	2.65 ± 1.01	.10	3.49 ± 2.21	2.28 ± 0.36	.10
3. Relationships with parents, siblings & other relatives- communicating, visiting, helping	**5.30 ± 0.18	**4.39 ± 0.32	.06	5.30 ± 0.51	3.53 ± 0.89	.15	5.31 ± 0.21	4.60 ± 0.36	.04
4. Having and rearing children	4.79 ± 0.21	4.47 ± 0.38	.01	4.65 ± 0.42	3.18 ± 0.75	.15	4.91 ± 0.25	4.61 ± 0.45	.00
5. Close relationships with spouse or significant other	5.02 ± 0.22	4.17 ± 0.38	.04	4.81 ± 2.91	2.91 ± 0.89	.17	5.19 ± 0.24	4.15 ± 0.43	.05
6. Close friends	5.45 ± 0.16	4.96 ± 0.28	.02	5.19 ± 0.43	4.26 ± 0.76	.06	5.54 ± 0.18	5.01 ± 0.32	.02
7. Helping and encouraging others, volunteering, giving advice	**5.62 ± 0.16	**4.10 ± 0.28	.20	5.73 ± 0.48	3.75 ± 0.84	.20	5.67 ± 0.17	4.16 ± 0.31	.19
8. Participating in organizations and public affairs	**5.05 ± 0.18	**3.62 ± 0.32	.11	4.86 ± 0.59	3.78 ± 1.05	.04	5.09 ± 0.19	3.61 ± 0.33	.16
9. Learning- attending school, improving understanding, getting additional knowledge	**4.46 ± 0.18	**3.51 ± 0.32	.10	4.11 ± 0.53	3.1 ± 0.93	.05	4.60 ± 0.20	3.47 ± 0.35	.09
10. Understanding yourself - knowing your assets and limitations - knowing what life is about	**5.44 ± 0.16	**4.24 ± 0.29	.12	5.83 ± 0.31	4.1 ± 0.54	.31	5.40 ± 0.19	4.16 ± 0.35	.11
11. Work - job or in home	*4.75 ± 0.17	*3.93 ± 0.30	.05	4.21 ± 0.41	3.61 ± 0.77	.03	4.88 ± 0.21	4.01 ± 0.36	.05
12. Expressing yourself creatively	**5.17 ± 0.18	**4.07 ± 0.32	.11	5.3 ± 0.51	2.9 ± 0.81	.27	5.16 ± 0.20	4.34 ± 0.37	.10
13. Socializing - meeting other people, doing things, parties, etc.	**5.09 ± 0.19	**3.66 ± 0.33	.13	5.21 ± 0.47	3.10 ± 0.82	.22	5.01 ± 0.21	3.93 ± 0.38	.10
14. Reading, listening to music, or observing entertainment	**5.81 ± 0.14	**4.71 ± 0.24	.14	6.24 ± 0.36	4.13 ± 0.63	.32	5.74 ± 0.16	4.81 ± 0.28	.10
15. Participating in active recreation	*4.40 ± 0.20	*3.42 ± 0.35	.10	4.61 ± 0.50	3.48 ± 0.87	.10	4.31 ± 0.22	3.54 ± 0.41	.03
16. Independence, doing for yourself	**5.15 ± 0.16	**3.59 ± 0.29	.20	5.59 ± 0.39	3.37 ± 0.70	.30	5.10 ± 0.19	3.59 ± 0.34	.20

×Partial eta squared values: Small = .01; Medium = .09; Large = .25

* = *p* ≤ 0.05; ** *p* ≤ 0.01

^a^The maximum total score is 112. ^b^ Individual items are scored on a 7-point scale

**Table 3:** Descriptive data for quality of life scores across gender and separately for men and women who own a hearing service dog and those on a waiting list: Data reported is means ± standard error means and partial eta squared (η_p_^2^) data

	Across Gender			Men			Women		
	With Dog (*n* = 111)	Waiting List (*n* = 30)	Power × (η_p_^2^)	With Dog (*n* = 19)	Waiting List (*n* = 13)	Power (η_p_^2^)	With Dog (*n* = 92)	Waiting List (*n* = 17)	Power (η_p_^2^)
Total Quality of Life	80.61 ± 1.61^a^	75.5 ± 3.17	.02	76.86 ± 5.19	69.96 ± 6.27	.02	81.77 ± 1.57	77.65 ± 3.67	.01
1. Material comforts home, food, conveniences, financial security	5.38 ± 0.11^b^	5.36 ± 0.23	.00	4.79 ± 0.35	5.31 ± 0.41	.03	5.55 ± 0.12	5.22 ± 0.27	.01
2. Health - being physically fit and vigorous	*4.74 ± 0.15	*3.99 ± 0.30	.03	4.69 ± 0.41	3.84 ± 0.49	.06	4.76 ± 0.16	4.06 ± 0.38	.03
3. Relationships with parents, siblings & other relatives- communicating, visiting, helping	4.88 ± 0.16	5.33 ± 0.32	.01	4.47 ± 0.39	4.92 ± 0.48	.02	5.00 ± 0.18	5.45 ± 0.42	.01
4. Having and rearing children	5.45 ± 0.16	5.52 ± 0.32	.00	4.52 ± 0.50	4.70 ± 0.61	.00	5.72 ± 0.16	5.73 ± 0.41	.00
5. Close relationships with spouse or significant other	5.35 ± 0.18	5.67 ± 0.35	.00	4.42 ± 0.57	5.46 ± 0.69	.04	5.59 ± 0.17	5.52 ± 0.41	.00
6. Close friends	5.56 ± 0.13	5.18 ± 0.27	.01	4.63 ± 0.41	4.77 ± 0.50	.00	5.81 ± 0.14	5.15 ± 0.32	.03
7. Helping and encouraging others, volunteering, giving advice	5.61 ± 0.13	5.35 ± 0.26	.01	4.99 ± 0.41	5.10 ± 0.5	.00	5.78 ± 0.13	5.34 ± 0.31	.02
8. Participating in organizations and public affairs	4.43 ± 0.19	3.80 ± 0.37	.02	4.63 ± 0.51	3.69 ± .061	.05	4.38 ± -.20	3.92 ± 0.47	.01
9. Learning- attending school, improving understanding, getting additional knowledge	*4.78 ± 0.17	*4.00 ± 0.34	.03	4.99 ± 0.46	3.23 ± 0.56	.17	4.76 ± 0.18	4.52 ± 0.43	.00
10. Understanding yourself - knowing your assets and limitations - knowing what life is about	5.48 ± 0.15	4.86 ± 0.29	.03	5.20 ± 0.45	3.77 ± 0.54	.12	5.61 ± 0.15	5.41 ± 0.34	.00
11. Work - job or in home	*4.96 ± 0.17	*4.20 ± 0.34	.03	5.16 ± 0.47	3.84 ± 0.58	.10	4.92 ± 0.18	4.46 ± 0.42	.01
12. Expressing yourself creatively	4.76 ± 0.17	4.94 ± 0.33	.00	4.84 ± 0.43	4.69 ± 0.51	.00	4.75 ± 0.18	5.11 ± 0.43	.01
13. Socializing - meeting other people, doing things, parties, etc.	3.88 ± 0.18	3.56 ± 0.36	.00	3.63 ± 0.43	3.01 ± 0.53	.03	3.96 ± 0.21	3.82 ± 0.47	.00
14. Reading, listening to music, or observing entertainment	5.06 ± 0.17	4.97 ± 0.35	.00	5.10 ± 0.42	4.77 ± 0.51	.01	5.01 ± 0.19	5.11 ± 0.45	.00
15. Participating in active recreation	4.51 ± 0.18	3.75 ± 0.36	.03	4.83 ± 0.43	3.70 ± 0.53	.09	4.34 ± 0.20	3.88 ± 0.46	.01
16. Independence, doing for yourself	*5.72 ± 0.15	*4.92 ± 0.30	.04	5.99 ± 0.39	5.10 ± 0.48	.06	5.64 ± 0.16	4.88 ± 0.38	.03

×Partial eta squared values: Small = .01; Medium = .09; Large = .25

* = *p* ≤ 0.05; ** *p* ≤ 0.01

^a^The maximum total score is 112. ^b^ Individual items are scored on a 7-point scale

## Results

### Age and Gender

To assess whether there was a significant difference in age and gender between those clients who had acquired a service dog and those who were on a waiting list control Chi square analyses were conducted. There was a significant difference in age between the two groups for those on the physical service dog database (χ^2^ = 11.91 df = 4, *p* = .01), more 18–25 year olds were waiting to acquire a dog, whereas more 35–35 year olds had already acquired a physical service dog. There was no significant difference in gender between the two groups for those on physical service dog database (*p <* 0.05; Table 1).

There was no significant difference in age between the two groups for those on hearing service dog database (*p <* 0.05). There was a significant difference in gender between the two groups for those on the hearing service dog database, more females had acquired a hearing dog than were on the waiting list control (χ^2^ = 9.25 df = 1, *p* = .002).

### Physical Service Dogs

#### Total Quality of Life

There was a significant effect of Service Dog status on total Quality of Life Scores, when controlling for age and gender; *F*(1, 95) = 23.11, *p* = .000. Those with a service dog scored significantly higher Quality of Life than those waiting for a service dog, with large effect size (Table 2).

#### Individual Quality of Life Items

Those with service dog scored significantly higher than those waiting for a service dog, when controlling for age and gender, on the following items: (Q1) Material Comforts: *F*(1, 95) = 4.72, *p* = .03; (Q2) Health: *F* = 8.88, *p* = .004; (Q3) Relationship with relatives: *F* = 5.76, *p* = .01; (Q7) Helping/Volunteering: *F* = 22.24, *p* = .000; (Q8) Participating in organisations: F = 14.86, *p* = .004; (Q9) Learning: *F* = 6.24, *p* = .01; (Q10) Understanding self: *F* = 12.24, *p* = .001; (Q11) Working: *F* = 5.13, *p* = .02; (Q12) Creative expression: *F* = 8.45, *p* = .005; (Q13) Socialising: *F* = 13.61, *p* = .000; (Q14) Active reaction: *F* = 5.54, *p* = .02; (Q15) Reading/Music: *F* = 14.57, *p* = .000; (Q16) Independence: *F* = 20.73, *p* = .000. There was no significant effect on: (Q4) Having or rearing children, (Q5) Close relationship with spouse or others and (Q6) Close friends (*F*s < 3.50 *ps* > .05). However, comparison of effect sizes across men and women indicates that owning a service dog improves satisfaction of ‘having or rearing children’ greater in men than it does women (Table 2). Indeed, in general effect sizes were greater for men than women, with the exception of helping/volunteering, participating in organisation and learning, which were greater for women.

### Hearing Service Dogs

#### Total Quality of Life

There was a potential trend to higher total Quality of Life scores for individuals who had a hearing service dog, compared to those on the waiting list, when controlling for age and gender (see Table 3), but this was not statistically significant *F*(1, 140) = 2.01, *p* = .15.

#### Individual Quality of Life Items

Those with a hearing service dog scored significantly higher than those waiting for a hearing service dog, when controlling for age and gender, on the following specific items: (Q2) Health: *F*(1, 140) = 4.63, *p* = .03; (Q9) Learning: *F* = 3.82, *p* = .05; (Q11) Working: *F* = 3.73, *p* = .05, and (Q16) Independence: *F* = 5.30, *p* = .02. There was a trend for higher scores on two items, that approached statistical significance: (Q10) Understanding self: *F* = 3.41, *p* = .06; and (Q14) Active recreation: *F* = 3.31, *p* = .06. There was no significant effect of hearing dog status on the other items (all *Fs* < 2.1, *ps* > .05). As with the physical service dog group, effect sizes were generally greater in men compared to women (Table 3). In particular men showed a greater effect of service dog ownership than women on understanding yourself. In contrast to the physical service dog group effect sizes were stronger in men compared to women for participating in organisations and learning.

## Discussion

With the aim of gathering initial data to investigate whether living with a trained service dog significantly affects perceptions of quality of life we analysed questionnaire responses from individuals on the Dogs for Good (physical service dog) and Hearing Dogs for Deaf People (hearing service dogs) database who lived with a service dog, and those who were waiting to receive a service dog (waiting list control). The results show, that when controlling for age and gender, individuals who owned a service dog scored higher on items on the quality of life scale than individuals on the waiting list control. These effects were more evident for individuals in the physical service dog group than the hearing service dog group.

Scores for total quality of life were higher for both client groups if they were living with a service dog as opposed to waiting to receive a dog. However, this effect was only statistically significant in the physical service dog group comparisons, not the hearing service dog group comparisons. Indeed, dog status (with, or waiting for, a service dog) significantly affected all except three items (having/rearing children, close relationship with spouse/others and close friends) on the quality of life scale for those with a physical service dog. The items which dog status did not affect were those related to having close relationships with others (friends, children, spouse). This suggests that owning a service dog (for physical or hearing impairments) does not affect personal relationships. This is in contrast to studies with pet dogs which have indicated that dog ownership is associated with better family relationships [28, 29]. It is possible that this is due to the fact that a service dog has been trained to focus specifically on the individual with a disability.

However, owning a physical service dog was associated with higher scores on ‘understanding yourself’, and questions surrounding interactions with others. This suggests that service dog ownership may be related to improved wider social networks (not family/close friends), which may increase an individual’s understanding of themselves. Indeed, individuals who owned a service dog showed greater satisfaction with their socializing, participating in organizations, helping and encouraging others and visiting and helping relatives. This result is congruent with previous studies investigating the effect of service dogs on social acknowledgement and facilitation [17, 20, 30]. It has been found that the presence of a service dog can increase social acknowledgement and contact from strangers to owners who are in wheelchairs [20]. Socially uncomfortable behaviours, such as gaze avoidance and path avoidance, decrease in the presence of a service dog [31]. In contrast, those who owned a hearing service dog did not show significantly higher satisfaction with items surrounding social activities, or understanding themselves. This may be because those who are eligible to receive a hearing dog are less likely to feel stigmatized or socially excluded as their disability may be less initially apparent than those with an overt physical disability, and as such the dog has reduced impact on this group. Another consideration, for future studies, is to record the frequency in which the two client groups use their dog in social situations, and the nature of the interactions between the dogs, their owner and other people to assess for mediators of this effect.

Participants in both groups (physical service dog and hearing service dog) had higher satisfaction on items relating to independence, learning, working and health (physically fit and vigorous), than those who did not yet own a service dog. This increased independence could bring substantial economic savings to health and care services; enabling individuals to complete tasks without assistance may also reduce the strain on often over-stretched community based support services [32] and increase the potential for individuals to contribute to the economy. Furthermore, by improving an individual’s capacity to actively engage in the community may not only bring direct benefits to society (e.g. by enhancing social participation), but may also bring further life-enhancing benefits (e.g. improve self-esteem). Evidence of greater independence in service dog owners are consistent with previous research which suggests that service dogs increase an individual’s confidence, independence and self-esteem [33–35]. With regards to working and learning, previous studies have suggested that the presence of a dog can improve children’s performance on a range of cognitive and perceptual tasks [36–38], but there has been little report of whether the same applies to adults and their service dog. Again, this finding is worthy of further investigation and could be related to considerable economic saving and increased societal functioning in terms of enabling individuals with disabilities to partake in paid job roles. Improved perceptions of physical health with service dog owners may be related to improved perceptions on items relating to personal fulfilment (e.g. independence, learning, engaging in recreational activities) and active interactions with others (e.g. socializing). By leading a fulfilling life, it is possible that participants believe that their health is not holding them back, and hence they report greater health satisfaction. In other words, the person feels able to cope (see [20]), and feelings of coping are directly related to reported health and quality of life [9]. Another possible explanation is that those with service dogs are more likely to go outdoors, which has been shown to improve health [39, 40], as well as meet others through dog walking; another factor shown to improve wellbeing [41, 42]. However, it is possible that those who are awaiting a service dog report poorer quality of life because they are anticipating the improvement that they believe a service dog will bring. This may result in a negative contrast in their self-perceived quality of life (i.e. they are lacking something which they might soon have which they anticipate will improve their life) or they may be holding off engaging in new health promoting activities until they receive a dog. Therefore, it is plausible that those who are on a waiting list to get a service dog may score lower on quality of life to those who are similarly living with disability who are not awaiting a dog. Nonetheless, it may also be that individuals are holding off taking up activities because without the dog they lack the psychological (e.g. confidence) or physical ability to do so. Future research using a three-participant cohort group (with service dog, on waiting list for a service dog, able to receive a service dog but not wishing to do so) could be used to further explore this possibility.

This is the first study known to the authors to suggest that owning a physical service dog increases pleasure and/or ability to partake in recreational activities, as evidenced through scores on items such as ‘Expressing yourself creatively’ and ‘Reading, listening to music, or observing entertainment’. This may prove an important point for future studies to focus on as increased engagement in activities may bring positive impact on psychological and physical health. Furthermore, the ability to engage in a range of activities, feelings of increased confidence [33–35] and confidence in the service dog during times of need [16] may further explain why those with a physical service dog reported greater satisfaction in relation to ‘Understanding yourself’.

This research represents an important first documentation of the effect that acquiring a service dog has on quality of life, whilst controlling for age and gender. Although not definitive, the study provides support for larger scale investigations into the impact of service dogs on quality of life, which could utilise more objective measures of quality of life, including physiological assessments of health and use of health and support services. Additionally, although the sample size was too small to conduct reliable statistical comparisons across the genders, reports of effect size shows some possibly interesting gender differences in the benefits of acquiring a service dog. In general, men showed greater quality of life satisfaction in the service dog group compared to the waiting list control than their female counterparts. Future large scale studies would be needed to assess the significance of these differences.

The findings of this study should be considered in light of its limitations. The study had a low response rate, with less than half of those contacted returning the completed questionnaire. In particular, response rates were low for those in the hearing dog group. Given that the effects of service dog ownership were less profound in this cohort it could be that this group experience less benefits (and have lower expectations of the benefits in the control group) and therefore were less motivated to take part in the study. The response rates may have increased if more than one reminder message had been sent out to those contacted. However, we chose not to administer multiple reminders in order to protect this potentially vulnerable participant group from additional stressors. It should also be noted that this this study did not record for how long the individual had owned the dog for. It would be interesting for future studies to assess whether the benefits of service dogs are evidenced immediately, or whether the benefits have a cumulative effect over time. Additionally, we did not record whether the participants owned any other pets at the time. Furthermore, whilst this study controlled for the effects of age and gender it would be useful for future studies to record and control for other demographic statuses, including occupation, and living arrangement.

## Conclusions

This study identifies new ways in which a service dog can impact and enhance the lives of people living with a physical and hearing disability. These improvements provide evidence that service dogs not only improve overall independence of their owners, but also enhance their personal fulfilment. Owning a service dog did not affect satisfaction with close relationships in this study. These findings have significant implications for defining the wider value of service dogs and for informing the development of future large scale, controlled trials to further evaluate how service dog ownerships relates to improved wellbeing.
